# An inactivated recombinant rabies virus displaying the Zika virus prM-E induces protective immunity against both pathogens

**DOI:** 10.1371/journal.pntd.0009484

**Published:** 2021-06-04

**Authors:** Hongli Jin, Cuicui Jiao, Zengguo Cao, Pei Huang, Hang Chi, Yujie Bai, Di Liu, Jianzhong Wang, Na Feng, Nan Li, Yongkun Zhao, Tiecheng Wang, Yuwei Gao, Songtao Yang, Xianzhu Xia, Hualei Wang

**Affiliations:** 1 Key Laboratory of Zoonosis Research, Ministry of Education, College of Veterinary Medicine, Jilin University, Changchun, China; 2 Key Laboratory of Jilin Province for Zoonosis Prevention and Control, Institute of Military Veterinary, Academy of Military Medical Sciences, Changchun, China; 3 College of Veterinary Medicine, Jilin Agricultural University, Changchun, China; Instituto Butantan, BRAZIL

## Abstract

The global spread of Zika virus (ZIKV), which caused a pandemic associated with Congenital Zika Syndrome and neuropathology in newborns and adults, prompted the pursuit of a safe and effective vaccine. Here, three kinds of recombinant rabies virus (RABV) encoding the prM-E protein of ZIKV were constructed: ZI-D (prM-E), ZI-E (transmembrane domain (TM) of prM-E replaced with RABV G) and ZI-F (signal peptide and TM domain of prM-E replaced with the region of RABV G). When the TM of prM-E was replaced with the region of RABV G (termed ZI-E), it promoted ZIKV E protein localization on the cell membrane and assembly on recombinant viruses. In addition, the change in the signal peptide with RABV G (termed ZI-F) was not conducive to foreign protein expression. The immunogenicity of recombinant viruses mixed with a complex adjuvant of ISA 201 VG and poly(I:C) was tested in BALB/c mice. After immunization with ZI-E, the anti-ZIKV IgG antibody lasted for at least 10 weeks. The titers of neutralizing antibodies (NAbs) against ZIKV and RABV at week 6 were all greater than the protective titers. Moreover, ZI-E stimulated the proliferation of splenic lymphocytes and promoted the secretion of cytokines. It also promoted the production of central memory T cells (TCMs) among CD4+/CD8+ T cells and stimulated B cell activation and maturation. These results indicate that ZI-E could induce ZIKV-specific humoral and cellular immune responses, which have the potential to be developed into a promising vaccine for protection against both ZIKV and RABV infections.

## Author summary

There is no approved vaccine for Zika virus (ZIKV) disease. Many research efforts are ongoing, e.g., inactivated, DNA, or viral vector vaccines. However, due to the complexity of the pathogenesis and immunology of ZIKV, new ideas about vaccine development still need to be considered. Rabies virus (RABV) vectored vaccines have been developed for many viruses, such as against Lassa virus, canine distemper virus, Middle East respiratory syndrome coronavirus, and filovirus, based on the advantages of the vector. In this study, three recombinant RABVs expressing ZIKV prM-E, named ZI-D, ZI-E and ZI-F, are described. Since ZI-D and ZI-E could express foreign proteins successfully, the author evaluated the immunogenicity of ZI-D and ZI-E mixed with a complex adjuvant of ISA 201 VG and poly(I:C) in BALB/c mice. The study demonstrates that ZI-E induced mice to produce NAbs against both RABV and ZIKV and elicited specific cellular immune responses. The authors believe that the ZI-E vaccine based on the RABV vector has the potential to prevent ZIKV and RABV infections. It has the potential to be used in ZIKV-RABV binary vaccines in areas where both ZIKV and RABV are threats.

## Introduction

Zika virus (ZIKV), which belongs to the family *Flaviviridae* and the genus *Flavivirus*, is an arbovirus and was first isolated from a febrile rhesus macaque in Uganda in 1947 [1]. In May 2015, a large-scale outbreak of ZIKV disease occurred in Brazil, showing a trend of spread and cross-border transmission. According to a World Health Organization (WHO) report, as of July 2019, a total of 87 countries and territories had cases of infection [2]. Fever, rash, arthralgia and conjunctivitis are the main clinical symptoms, and the disease rarely causes death in adults, but it can cause a high risk of Congenital Zika Syndrome (CZS, a combination of severe neurological anomalies) in newborn babies after infection of pregnant women [3,4].

Currently, there is no approved vaccine or effective treatment for ZIKV disease. However, intensive research efforts in this field have been ongoing. Using other viruses as vectors to express the main structural proteins of ZIKV is a strategy to develop ZIKV vaccines, including the recombinant replication-deficient adenovirus [5], the rhesus adenovirus serotype 52 vectored vaccine [6], the attenuated poxvirus vectored vaccine [7], and the attenuated recombinant vesicular stomatitis virus vectored vaccine [8]. However, due to the complexity of the pathogenesis and immunology of ZIKV, there are many uncertainties associated with the development of vaccines [9,10]. New ideas and methods still need to be further considered [11]. Several important characteristics recommend rabies virus (RABV) vectors as vaccine delivery platforms. First, RABV can be replicated and transcribed with high efficiency in target cells to produce abundant virus-specific proteins that favor the packaging of the recombinant virus. Furthermore, since few people possess serum antibodies against RABV, preexisting RABV seropositivity is negligible [12]. Using reverse genetic manipulation techniques, novel RABV vectored vaccines have been developed for many viruses based on the advantages of the vector [12], such as against Lassa virus (LASV) [13], canine distemper virus (CDV) [14], Middle East respiratory syndrome coronavirus (MERS-CoV) [15] and filovirus [16].

The E protein, a structural protein, is the only envelope glycoprotein of ZIKV and plays important roles in the molecular recognition, pathogenesis and immune process of the virus. E protein containing neutralizing epitopes has been targeted in ZIKV vaccine research [17–20]. PrM-E can be cleaved into the prM and E proteins by host cell signal peptidase [21], and prM plays a critical role in folding the E protein [18]. In this study, a recombinant RABV expressing ZIKV prM-E protein was constructed based on a RABV reverse genetic operating system [22]. Humoral and cellular immune responses were stimulated after intramuscular immunization in mice.

## Materials and methods

### Ethics statement

All of the mice were treated in accordance with the Chinese ethical guidelines for the welfare of laboratory animals (GB 14925–2001). The study was approved by the Animal Welfare and Ethics Committee of the Institute of Veterinary Medicine of the Military Academy of Sciences (Laboratory Animal Care and Use Committee Authorization permit number JSY-DW-2018-02).

### Viruses, cells and antibodies

The recombinant viruses were grown in suspended baby hamster kidney (BHK-21) cells that were maintained in CD BHK-21 Production Medium (Thermo Fisher Scientific, Waltham, MA, USA) with 1% fetal bovine serum (FBS, Gibco, Grand Island, NY, USA) at 37°C and 120 rpm. The ZIKV ZKC2 2016 strain was cultivated in C6/36 cells (10% MEM with 10% FBS) at 27°C. BSR cells, which are a cloned cell line derived from BHK-21 cells, were maintained in Dulbecco’s modified Eagle’s medium (DMEM, Gibco, Grand Island, NY, USA) that contained 5% FBS. NA cells were maintained in DMEM that contained 10% FBS.

A fluorescein isothiocyanate (FITC)-conjugated monoclonal antibody (mAb) against RABV N protein (800–092) was purchased from Fujirebio (Melvin, PA, USA). An anti-RABV G mAb (MAB8727) was purchased from Millipore (Billerica, MA, USA). A rabbit anti-ZIKV E polyclonal antibody (GTX133314) was purchased from GeneTex (Alton PkwyIrvine, CA, USA). TRITC-conjugated goat anti-mouse IgG (T5393) was purchased from Sigma (St. Louis, MO, USA). FITC-conjugated goat anti-rabbit IgG (ab6717), horseradish peroxidase (HRP)-conjugated goat anti-mouse IgG (ab6789), HRP-conjugated goat anti-rabbit IgG (ab6721), donkey anti-mouse IgG H&L (ab39593, 10 nm gold) and donkey anti-rabbit IgG H&L (ab105296, 18 nm gold) were purchased from Abcam (Cambridge, MA, USA).

### cDNA construction of vaccine vectors

The reverse genetic operating system of the vaccine vector RABV SRV9 has been described previously [22]. In this vector, an exogenous gene expression component “PE-PS-*BsiW* I-*Pme* I” was introduced, and foreign proteins could be expressed through the two restriction sites “*BsiW* I-*Pme* I”. Full-length ZIKV prM-E cDNA was retrieved from GenBank (Accession: KX601168.1). The TM or signal sequence region of ZIKV prM-E was replaced by the corresponding regions of the RABV SRV9 strain (Accession: KX601168.1). Foreign genes were optimized for mammalian cells and synthesized by Sangon Biotech (Shanghai, China), and genes were introduced into the vector RABV SRV9 using *BsiW* I and *Pme* I restriction digestion sites. Three kinds of full-length viral cDNA containing ZIKV prM-E were constructed, namely ZI-D (full-length prM-E), ZI-E (full-length prM-E with TM region replaced by the corresponding region of SRV9) and ZI-F (full-length prM-E with the signal sequence and TM replaced by SRV9).

The recombinant viruses were recovered as described previously [22]. Briefly, Lipofectamine 3000 Transfection Reagent (Invitrogen, Carlsbad, CA, USA) was used to cotransfect the full-length viral cDNA along with the helper plasmids (encoding the RABV N, P, G and L proteins, respectively) into BSR cells. Seven days later, the supernatants were harvested and analyzed by immunostaining for RABV N.

### Immunofluorescence analysis (IFA)

For detection or titration of RABV, NA cells were seeded in 96-well plates and infected with tenfold serial dilutions of viruses (50 μl/well). Each dilution was performed in quadruplicate. Forty-eight hours later, the cells were fixed with 80% cold acetone, and a FITC-conjugated anti-RABV N mAb (1:200) served as the detection signal for RABV. Fluorescence was observed under a fluorescence microscope (Olympus, Tokyo, Japan). The titer of RABV was calculated according to the Reed-Muench method.

For confocal microscopy analysis, NA cells were seeded on confocal dishes and infected at an MOI of 1 with the different viruses. Forty-eight hours later, the cells were fixed with 4% paraformaldehyde (PFA) and permeabilized with 0.2% Triton X-100 or nonpermeabilized. After blocking with 1% BSA, the cells were incubated with an anti-RABV G mAb (1:100) and an anti-ZIKV E polyclonal antibody (1:100). Then, a TRITC-conjugated anti-mouse antibody (1:500) and a FITC-conjugated anti-rabbit antibody (1:300) were added to the corresponding primary antibodies. The cells were stained with 4,6-diamidino-2-phenylindole (DAPI) and imaged with a confocal microscope.

### Electron microscopy analysis

Recombinant RABVs were negatively stained with uranyl acetate and then examined under an electron microscope. For immunoelectron microscopy, after attachment to copper mesh, the samples were double stained with an anti-RABV G mAb (1:50) and a rabbit anti-ZIKV E polyclonal antibody (1:50). After they were washed with PBS, the samples were labeled with donkey anti-rabbit IgG (18 nm gold, 1:20) and donkey anti-mouse IgG (10 nm gold, 1:20). The stained samples were examined under an electron microscope.

### One-step growth curves

BHK-21 suspension cells in 30-ml volumes were infected with the recombinant viruses and RABV SRV9 at MOIs of 0.1, 0.5 and 1, and samples of 200 μl were harvested every 24 h. The samples harvested from 1–4 days post infection (dpi) were titrated in NA cells as described above.

### Virus purification

Recombinant RABV was inoculated into BHK-21 suspension cells at an MOI of 0.5 for large-scale purification. The supernatant was collected on the 2nd and 4th days and replaced with fresh medium. After centrifugation at 3000 rpm for 30 min, the viruses in the supernatants were precipitated with zinc acetate. After resuspension, the viruses were purified by ultracentrifugation through a 10-20-30-40-55% sucrose gradient for 1.5 h at 50,200 g. The viruses that remained in 30–40% sucrose were resuspended in STE (0.15 M NaCl, 0.001 M EDTA, 0.01 M Tris-base, pH = 7.4) and inactivated using beta-propiolactone (BPL) (Serva Electrophoresis GmbH, Heidelberg, Germany) at a 1:3000 dilution. After incubation at 4°C for 24 h, BPL was hydrolyzed at 37°C for 2 h. The protein concentrations of the inactivated viruses were measured using a BCA Protein Assay Kit (Pierce, Rockford, IL, USA) according to the manufacturer’s instructions.

### Sodium dodecyl sulfate (SDS)-polyacrylamide gel electrophoresis (PAGE) and western blot (WB)

Purified viruses were denatured in loading buffer at 100°C for 5 min. 30 μg of denatured proteins were separated by 10% SDS-PAGE. For total protein analysis, the gels were stained with Coomassie brilliant blue. For WB, the proteins separated by SDS-PAGE were transferred from the gels to nitrocellulose (NC) membranes (GE Healthcare, Little Chalfont, Buckinghamshire, UK) for immunoblot analysis. Polyclonal rabbit anti-ZIKV E antibodies (1:700) were used as the primary antibodies, and HRP-conjugated goat anti-rabbit IgG (1:5000) was used as the secondary antibody. Electrochemiluminescence (ECL) Western Blotting Substrate (Pierce, IL, USA) was added, and the bands were captured using a Tanon-5200 Chemiluminescent Imaging System (Tanon, Shanghai, China).

### Immunizations in mice

Female BALB/c mice (6–8 weeks) were randomly divided into 3 groups (n = 9/group) and vaccinated intramuscularly (IM) with ZI-D or ZI-E mixed with a complex adjuvant of ISA 201 VG (containing special mineral oil, Seppic, Paris, France) and poly(I:C) (Sigma, St. Louis, MO, USA). To ensure the immune effect, we chose twice the dose of references [15,16,23], i.e., 20 μg as the immune dose. Each milliliter of vaccine contains 55% volume ratio of ISA 201 VG and 200 μg of poly(I:C). Mice that received PBS were used as the controls. Nine mice from each group were boosted twice at 2-week intervals. The sera were collected at 0-, 2-, 5-, and 8-weeks post immunization (wpi) and heat inactivated at 56°C for 30 min.

### ELISA

Indirect ELISA was performed to detect the IgG titer or the ratio of IgG2a/IgG1 in serum. Purified ZIKV E protein from insect cells (MyBioSource, San Diego, CA, USA) was diluted in coating buffer (50 mM Na_2_CO_3_, pH 9.6) at a concentration of 0.5 μg/ml and then plated in 96-well microtiter plates (Corning-Costar, Corning, NY, USA) at a volume of 100 μl/well. The plates were incubated at 4°C overnight. After blocking with 5% nonfat milk, the plates were incubated with twofold serial dilutions of sera in blocking buffer. Next, HRP-conjugated goat anti-mouse IgG (H+L) (1:2000, BioWorld, St. Louis, MO, USA), IgG1, or IgG2a (1:2000, Southern Biotech, Birmingham, AL, USA) was added. Then, tetramethylbenzidine (TMB, Sigma, St. Louis, MO, USA) was added as an acidic buffer, and 2 M H_2_SO_4_ was used to stop color development. The absorbance was read at 450 nm using a microplate reader (Thermo Fisher Scientific, Waltham, MA, USA).

### Neutralization titer assay

A virus neutralization assay (VNA) for ZIKV was performed as previously described [11]. Briefly, twofold serial dilutions of heat-inactivated serum were mixed with equal volumes of ZIKV (500 PFU/ml). After incubation for 1 h at 37°C, 200 μl of the mixture was transferred to monolayer BHK cells in 12-well plates, and the cells were incubated for another 1 h. Then, the inoculum was removed and replaced with semisolid agarose medium. After incubation for four days, the cells were fixed and stained with crystal violet. The titers were determined by a standard 50% plaque reduction neutralization test (PRNT_50_).

A VNA for RABV was conducted using a fluorescent antibody virus neutralization (FAVN) test. Briefly, 100 μl volumes of threefold serial dilutions of serum were mixed with 100 TCID_50_ of CVS-11 stain in 96-well plates, and the mixtures were incubated for 1 h at 37°C. Then, NA cells were added and incubated for 48 h at 37°C. The cells were fixed with cold 80% acetone and stained with FITC-conjugated anti-RABV N antibodies (1:200). The positive/negative wells were determined under a fluorescence microscope, and the titers of RABV NAbs are expressed in IU/ml by comparison with a standard serum sample (0.5 IU/ml).

### Splenocyte proliferation assay

One week after the last immunization, three mice from each group were euthanized. Mouse spleens were collected and disrupted with the plunger of a syringe. The splenocyte suspensions were strained through a 100 μm mesh, and red blood cells were lysed by erythrocyte lysis buffer (Solarbio, Beijing, China). Then, 2.5×10^5^ splenocytes (suspended in complete RPMI 1640), along with purified ZIKV E antigen (10 μg/ml), were plated in 96-well plates for culture. After 44 h, 10 μl of commercial TransDetect Cell Counting Kit-8 (CCK-8) reagent (KeyGEN Biotech, Nanjing, China) was added to the cells, and the cells were cultivated for another 4 h. Then, the absorbance was measured at 450 nm using a microplate reader (Thermo Fisher Scientific, Waltham, MA, USA). The proliferation index (PI) was calculated as follows: (OD _stimulated cultures_–OD_unstimulated cultures_)/(OD_unstimulated cultures_−OD_control cultures_).

### Ex vivo IFN-γ and IL-4 ELISpot assay

A total of 5×10^5^ splenocytes (treated as in the splenocyte proliferation assay), along with purified ZIKV E antigen (10 μg/ml), were plated in 96-well ELISpot plates (MABTECH, Nacka, Sweden). After 24 h, the cells were incubated with a biotinylated IFN-γ or IL-4 antibody (1:1000) and later labeled with streptavidin-conjugated HRP (1:1000). TMB was used to develop spots, and SFCs were acquired using an ELISpot reader (Multispotreader Spectrum, AID, Strasberg, Germany).

### Measurement of cytokine levels in splenocyte culture supernatants

A total of 5×10^5^ splenocytes (treated as in the splenocyte proliferation assay) along with purified ZIKV E antigen (10 μg/ml) were plated in 96-well plates. After culture for 72 h, the supernatant was collected for the detection of cytokines using a Meso Scale Discovery (MSD) kit, and the data were measured with a Meso QuickPlex SQ120 (Meso Scale Diagnostics, Rockville, MD, USA).

### Cell-surface molecule staining

A total of 5×10^5^ splenocytes (treated as in the splenocyte proliferation assay) along with purified ZIKV E antigen (10 μg/ml) were plated in 96-well plates. After culture for 72 h, the cells were collected in PBS containing 2% FBS and 0.1% NaN_3_. Then, the cells were stained with 0.25 μg of FITC-conjugated anti-CD4, PE-conjugated anti-CD8, APC-conjugated anti-CD19, PE/Cy7-conjugated anti-CD69, APC-conjugated anti-CD44 or PerCP-Cy5.5-conjugated anti-CD62L (BD Biosciences, Franklin, CA, USA) antibodies for 30 min at 4°C. Data were acquired on a FACSCalibur flow cytometer (BD Biosciences, Franklin, CA, USA).

### Statistical analyses

All of the experiments were repeated three times, and the results are expressed as the mean ± SD. All statistical analyses were performed with Student’s t-test in GraphPad software, version 8.0.2. A p value of ≤0.05 was considered statistically significant.

## Results

### Construction of recombinant virus with three different strategies

**Fig 1:**
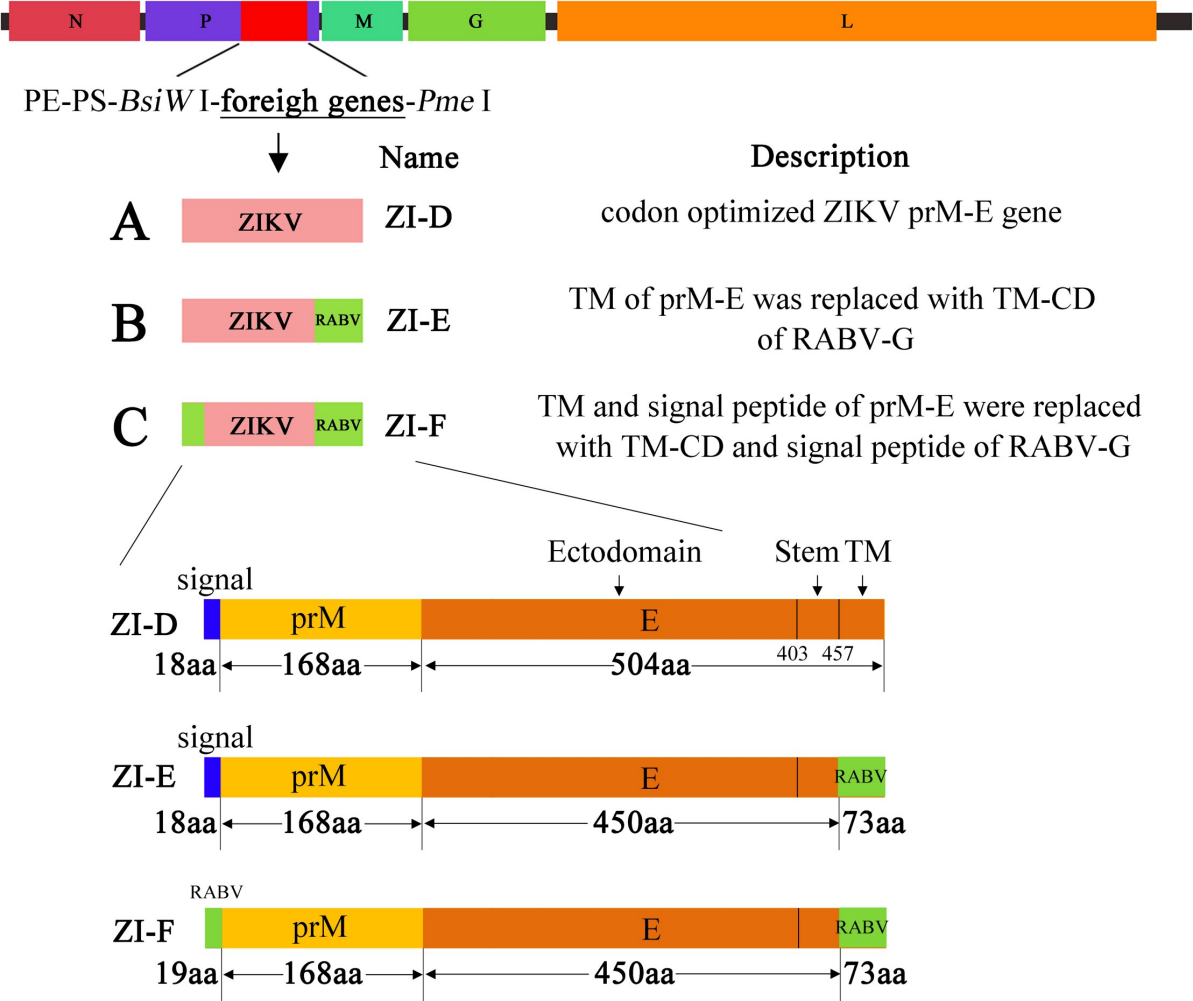
Schematic diagram for cDNA construction of vaccine vectors. Based on the reverse genetic operating system of the vaccine vector RABV SRV9, which contains the exogenous gene expression component, foreign genes were cloned between the P and M genes of full-length SRV9 using *BsiW* I and *Pme* I restriction digestion sites. (A) ZI-D: full-length prM-E gene of ZIKV. (B) ZI-E: full-length prM-E of ZIKV with the TM domain replaced by the TM-CD of RABV. (C) ZI-F: full-length prM-E of ZIKV with the signal sequence and TM domain replaced by the corresponding region of RABV.

**Fig 2:**
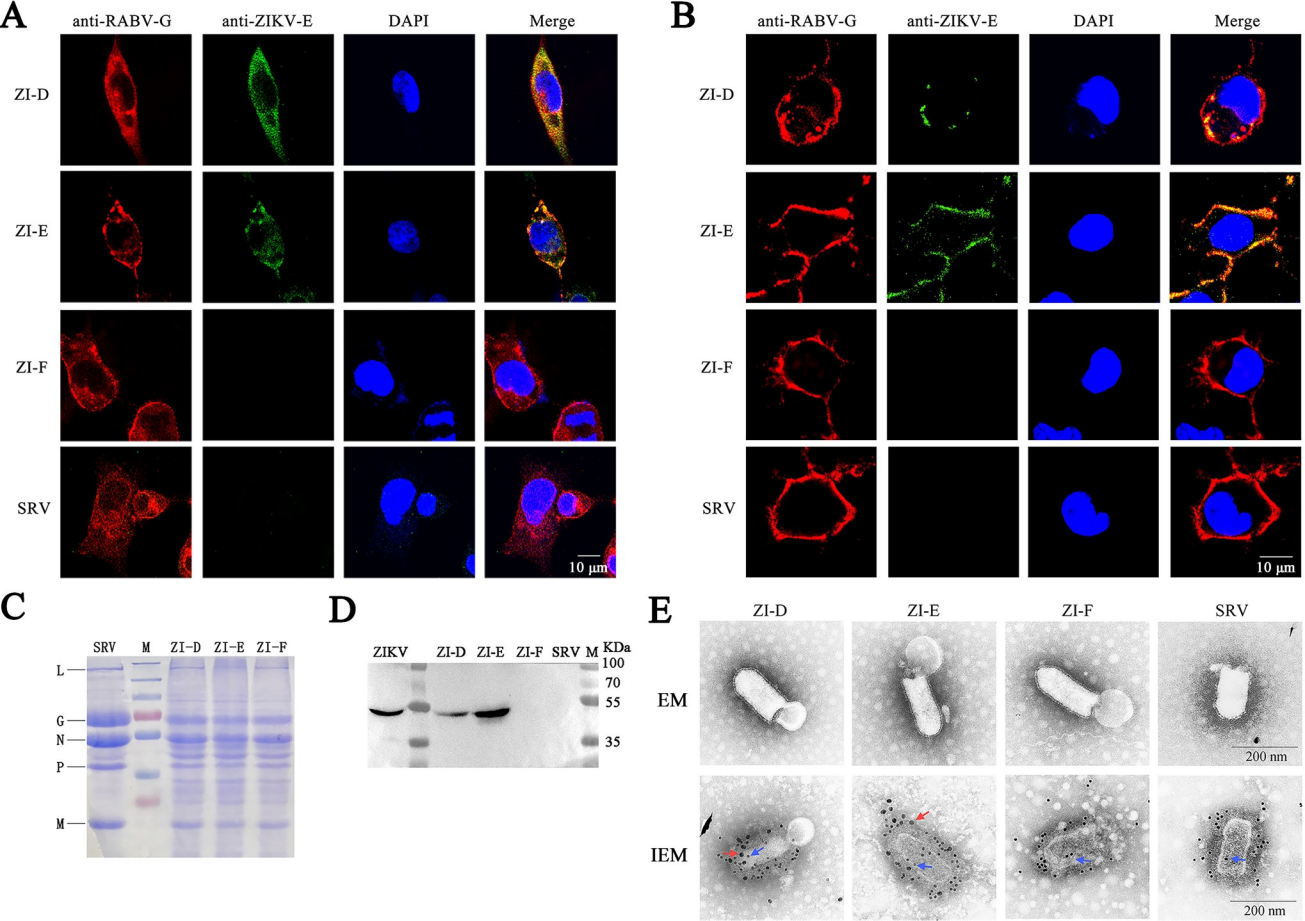
Identification of recombinant viruses. (A-B) Confocal microscopy analysis (6000×). After infection with ZI-D, ZI-E, ZI-F and RABV SRV9 control for 48 h, NA cells were permeabilized (A) or nonpermeabilized (B) and immunostained with antibodies against RABV G protein (red fluorescence) and ZIKV E protein (green fluorescence). Blue indicates DAPI-stained nuclei. Scale bars represent 10 μm. (C) Sucrose density gradient centrifugation-purified virions were analyzed by SDS-PAGE. (D) Detection of ZIKV E protein in sucrose-purified virions by WB. (E) Electron microscopy (EM) and dual-label immunogold electron microscopy (IEM) detection of recombinant viruses. 10 nm gold particles (blue arrow) were used to label RABV G, and 18 nm gold particles (red arrow) were used to label flavivirus E. Scale bars represent 200 nm.

To express the prM-E proteins of ZIKV, we generated recombinant RABV based on the SRV9 strain [22] with three strategies (Fig 1). ZI-D (codon-optimized ZIKV prM-E, Fig 1A), ZI-E (in which the transmembrane (TM) domain of prM-E (amino acids 451–504) was replaced with the TM-cytoplasmic domain (TM-CD) of RABV, Fig 1B) and ZI-F (in which the signal peptide and TM domain of prM-E were replaced with the signal peptide and TM-CD region of RABV, Fig 1C) were cloned into the RABV SRV9 vector using two unique restriction sites (*BsiW* I and *Pme* I) that flank a RABV transcription start/stop signal between the RABV P and M genes. All three recombinant viruses were successfully recovered. To verify the expression of foreign proteins, NA cells were infected at a multiplicity of infection (MOI) of 1 and immunostained with antibodies against RABV G and flavivirus E. The results showed that both RABV G and ZIKV E could be detected in ZI-D and ZI-E after the cells were permeabilized. However, in ZI-F, only RABV-G was expressed, and ZIKV E was not detected (Fig 2A). Moreover, to detect whether ZIKV E protein could be detected on the cell surface, nonpermeabilized cells were analyzed by confocal immunofluorescence. The results indicated that the ZIKV E protein was present on the surface of ZI-E-infected cells, while there was little ZIKV E protein present on the surface of ZI-D-infected cells (Fig 2B). No ZIKV E protein was detected in ZI-F-infected cells.

To detect the incorporation of foreign protein in the recombinant viruses, sucrose-purified virions from infected BHK cells were analyzed by SDS-PAGE and western blotting (WB). SDS-PAGE of purified ZI-D, ZI-E and ZI-F showed similar migration of proteins of the expected size for the RABV proteins compared with the RABV SRV9 controls (Fig 2C). However, ZIKV E protein (55 kDa) comigrated with RABV N protein (57 kDa) and was difficult to confirm. Therefore, incorporation of ZIKV E protein was detected by WB analysis. The results (Fig 2D) indicated that ZI-D and ZI-E incorporated ZIKV E protein in recombinant viruses with correct protein cleavage and that ZI-E was expressed at higher levels than ZI-D. There was no target band for ZI-F.

To detect whether the presence of foreign proteins affected the structure of the virus, the recombinant viruses were analyzed by electron microscopy. Fig 2E shows that typical-sized, bullet-shaped RABV particles could be seen in ZI-D, ZI-E and ZI-F. Furthermore, the viruses were analyzed by dual-label immunogold electron microscopy with anti-RABV G (10 nm gold particles) and anti-flavivirus E (18 nm gold particles). Both ZI-D and ZI-E were found to react with anti-RABV G and anti-flavivirus E antibodies, while ZI-F was labeled only by anti-RABV G antibodies (Fig 2E). The immunogold electron microscopy results were consistent with the fluorescence staining and WB results.

### Growth kinetics of the recombinant viruses

**Fig 3:**
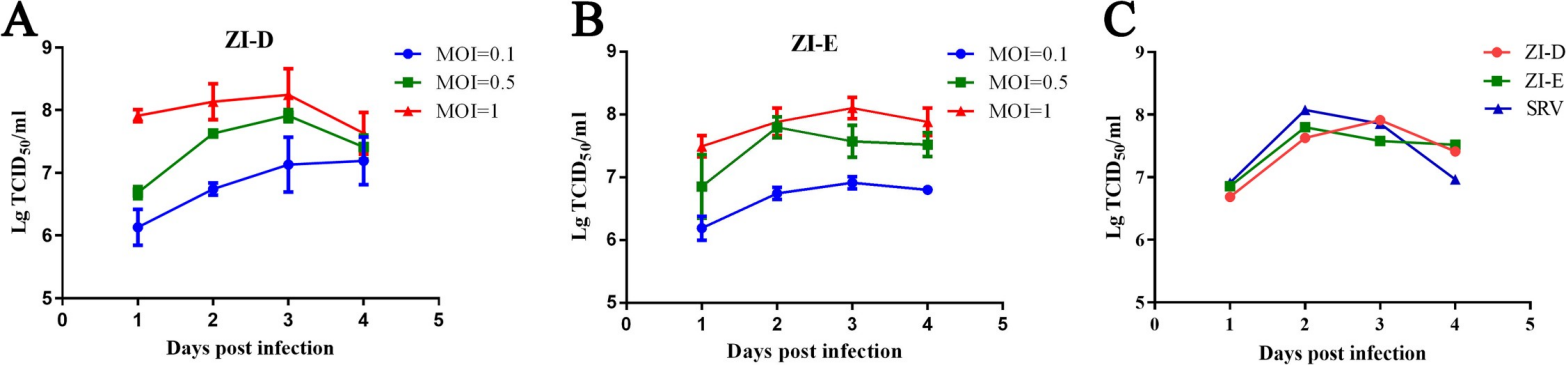
Growth curves of the recombinant virus. (A-B) BHK suspension cells were infected with ZI-D or ZI-E at an MOI of 0.1, 0.5 or 1. On days 1, 2, 3 and 4 after infection, samples were obtained, and the RABV titers were determined in NA cells. (C) RABV titer comparison of the recombinant virus with RABV SRV9 at an MOI of 0.5. The tests were repeated three times.

To identify whether foreign proteins would affect RABV growth kinetics, one-step growth curves of ZI-D and ZI-E were analyzed using BHK-21 suspension cells (Fig 3). The titers of the recombinant viruses all reached 10^8^ TCID_50_/ml, and the viral titer increased with increasing MOI (Fig 3A and 3B). Moreover, at an MOI = 0.5, there were no significant differences in growth abilities among recombinant viruses and RABV SRV9 (Fig 3C). The results indicated that the growth kinetics were not affected by foreign protein expression and that the recombinant viruses could be cultured in suspension, conducive to large-scale virus production.

### Recombinant viruses induce ZIKV-specific IgGs and neutralizing antibodies (NAbs)

**Fig 4:**
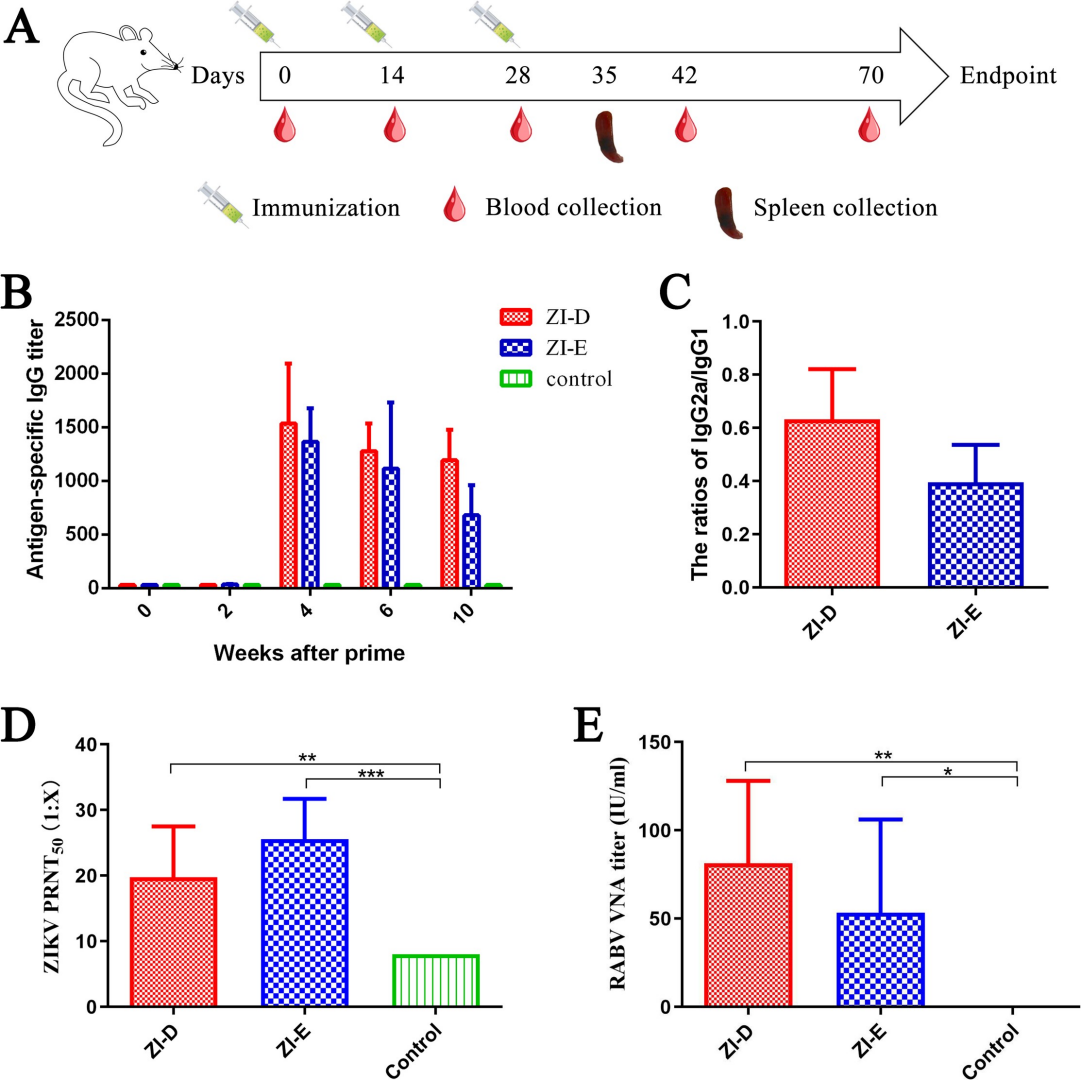
Immunization evaluation. (A) Immunization strategy. BALB/c mice (n = 9) were immunized IM with ZI-D or ZI-E (20 μg) mixed with a complex adjuvant of ISA 201 VG and poly(I:C). The mice were boosted twice at 2-week intervals. Mice that received PBS were used as controls. Blood samples (n = 6/group) were collected on days 0, 14, 28, 42 and 70. On the 35th day, mouse spleens (n = 3/group) were collected. (B) ZIKV E-specific IgG titers were assessed by indirect ELISA with the purified E protein and are displayed as the end-point dilution titers. (C) IgG2a/IgG1 ratios at week 6 as assessed by indirect ELISA with the purified E protein. (D) ZIKV NAb titer at week 6 as determined by a standard PRNT_50_. (E) RABV NAb titer at week 6 as determined by a FAVN test.

Based on the above identification results, we chose ZI-D/ZI-E as an immunogen. BALB/c mice were intramuscularly immunized with 20 μg of beta-propiolactone (BPL)-inactivated ZI-D or ZI-E, following a three-inoculation vaccination schedule (Fig 4A). Serum was collected, and the humoral immune response was analyzed periodically until week 10. Analysis of the levels of IgG against ZIKV E by enzyme-linked immunosorbent assay (ELISA) indicated that both inactivated ZI-D and ZI-E achieved appreciable IgG responses against ZIKV E (Fig 4B). Then, we examined the quality of this humoral response by IgG2a and IgG1 subisotype-specific ZIKV E ELISA at week 6 (Fig 4C). IgG2a/IgG1 ratios less than 1.0 indicated that ZI-D/ZI-E induced a T-helper (Th)2-biased response.

To predict protection against ZIKV, the development of neutralizing antibodies (NAbs) (Fig 4D) was quantified in the serum of mice at week 6. The results showed that most of the immunized mice generated protective titers (>10). Moreover, the titer of ZI-E was slightly higher than that of ZI-D, which might have been due to increased expression of the target protein of ZIKV.

In addition, RABV NAbs were evaluated using the World Organization for Animal Health (OIE) standard, which considers values >0.5 international unit (IU)/ml to be protective against RABV. By week 6, the concentrations in the immunized groups had all reached at least 23.38 IU/ml, much higher than 0.5 IU/ml (Fig 4C), indicating that the addition of ZIKV E to the RABV backbone did not compromise the ability of the backbone to generate protective RABV NAbs.

### Splenocyte proliferation and cytokine secretion by ex vivo restimulation

**Fig 5:**
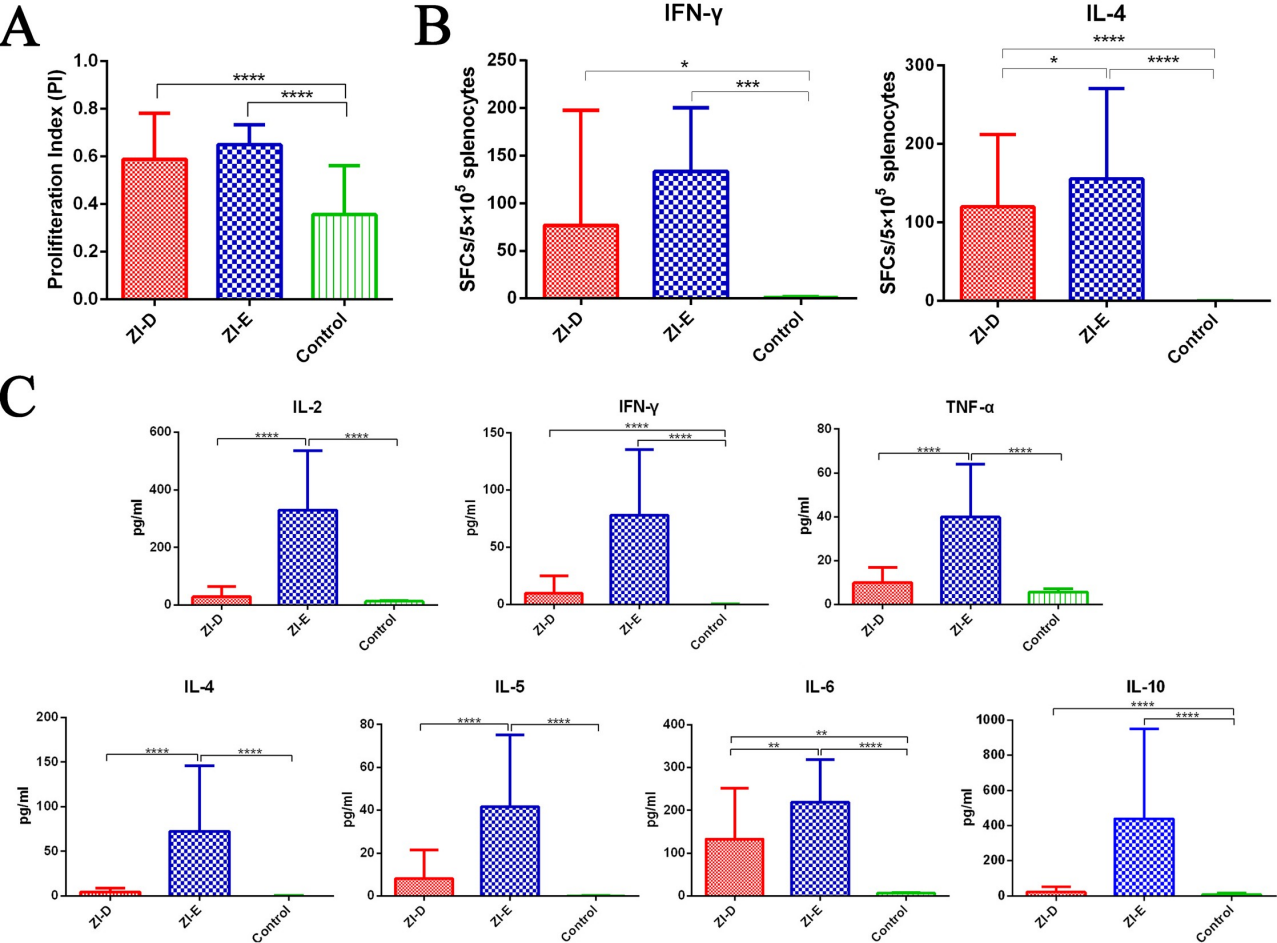
Splenocyte proliferation. One week after the last immunization, mouse spleens (n = 3/group) were collected, and splenocytes were restimulated with purified ZIKV E protein. (A) The proliferative index was detected using a CCK-8 assay. (B) The levels of IFN-γ and IL-4 secreted by splenocytes were quantified by ELISpot assay. (C) Cytokine levels in splenocyte culture supernatants were measured by MSD. Each sample was repeated three times. The data are expressed as the mean ± SD for each group. *p<0.05; **p<0.01; ***p<0.001; ****p<0.0001.

One week after the last immunization with ZI-D or ZI-E, an ex vivo splenocyte proliferation assay was performed to evaluate the effects of the vaccines on splenocyte proliferation responses (Fig 5A). After stimulation with the ZIKV E protein, splenocytes from mice in the immunized groups proliferated more efficiently than those from mice in the PBS control group.

Moreover, the capacity of splenocytes to produce interferon (IFN)-γ and interleukin (IL)-4 in response to antigens was quantified in vitro by enzyme-linked immunosorbent spot (ELISpot) assay (Fig 5B). Significantly elevated numbers of spot-forming cells (SFCs) for IFN-γ and IL-4 responses were detected in immunized mouse splenocytes, and ZI-E induced more robust IL-4 responses than ZI-D.

Next, we tested whether the immunized groups exhibited elevated secretion of cytokines. The levels of cytokines were detected in the supernatant of stimulated splenocytes from all of the groups (Fig 5C). The results showed that the levels of Th1 cytokines (IL-2, IFN-γ and TNF-α) and Th2 cytokines (IL-4, IL-5, IL-6 and IL-10) in the immunized groups were significantly higher than those in the PBS control group; in addition, the levels of these cytokines produced by ZI-E were higher than those produced by ZI-D. Moreover, the trends in the cytokines IFN-γ and IL-4 were in accordance with the findings of the ELISpot assay. Overall, the data above indicated that ZI-D and ZI-E could induce the secretion of a balance of Th1 and Th2 cytokines.

These data suggested that ZI-D and ZI-E evoked immune cell proliferation and elicited potent antigen-specific cellular immune responses in mice. Moreover, ZI-E had greater potential to induce cytokine production than ZI-D.

### Lymphocyte activation assays

**Fig 6:**
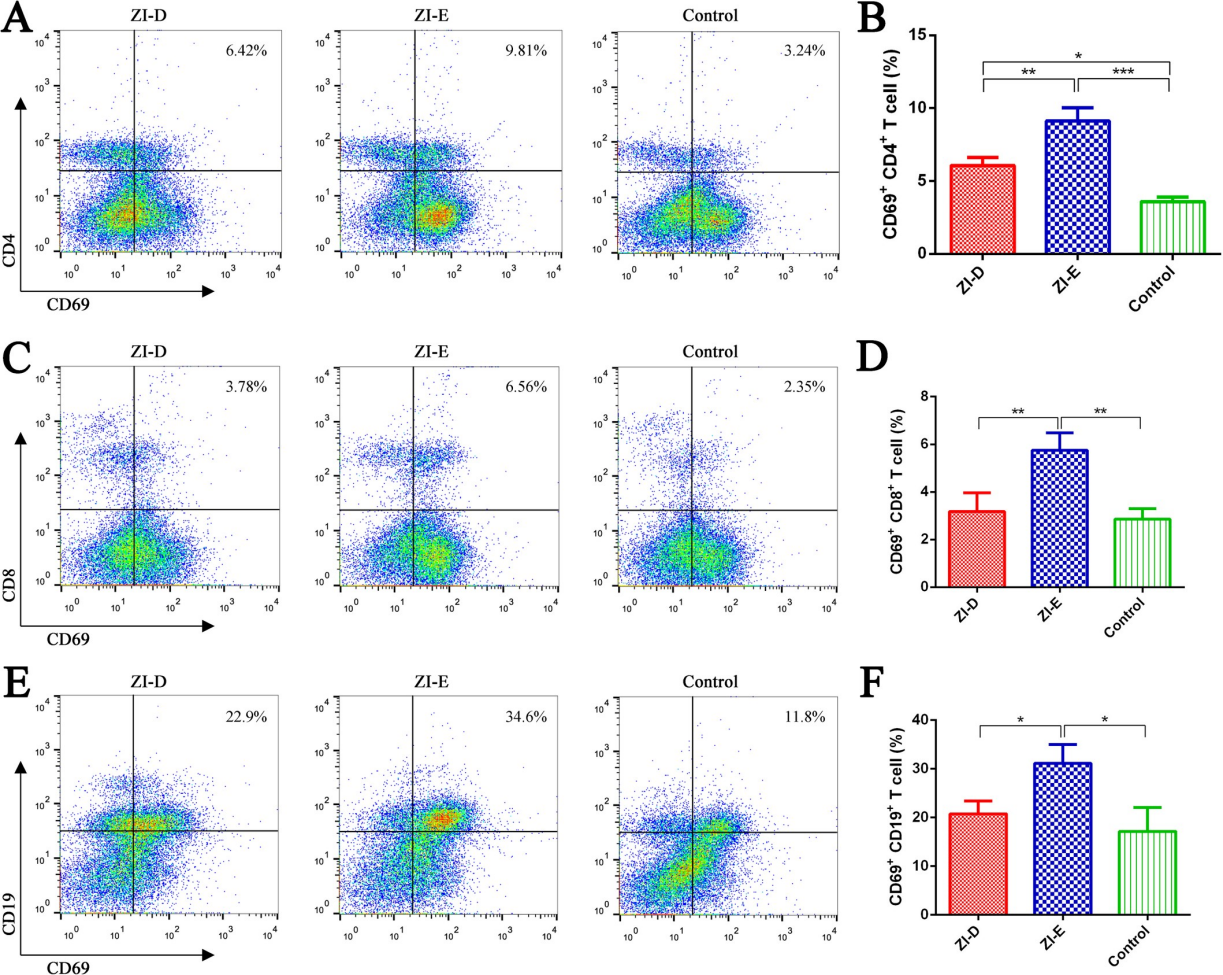
Lymphocyte activation. One week after the last immunization, mouse splenocytes (n = 3/group) were collected and restimulated with purified ZIKV E protein. The activation of lymphocytes, including CD4^+^ T cells (A-B), CD8^+^ T cells (C-D) and B cells (E-F), was evaluated by flow cytometry. (A, C, E) Representative flow cytometric plots of lymphocytes from each group. (B, D, F) Percentages of the indicated lymphocytes. The data are expressed as the mean ± SD for each group. *p<0.05; **p<0.01; ***p<0.001.

CD69 is a surface antigen present after T/B cell activation; therefore, we detected the proportion of CD69^+^ lymphocytes in the spleen by flow cytometry to analyze the activation of CD4^+^ and CD8^+^ T cells and B cells. The results showed that the proportions of CD4^+^CD69^+^ T cells (Fig 6A and 6B), CD8^+^CD69^+^ T cells (Fig 6C and 6D) and CD19^+^CD69^+^ B cells (Fig 6E and 6F) in the ZI-E group were all significantly higher than those in the ZI-D and PBS control groups, indicating that ZI-E with a complex adjuvant of ISA 201 VG and poly(I:C) could promote T cell and B cell activation.

**Fig 7:**
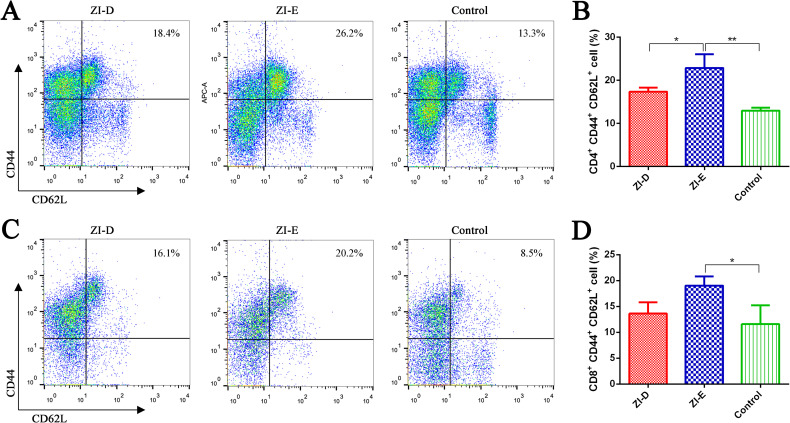
Proportion of TCMs. One week after the last immunization, mouse splenocytes (n = 3/group) were collected and restimulated with purified ZIKV E protein. The proportions of TCMs (CD44^+^CD62L^+^) among CD4^+^ (A-B) and CD8^+^ T (C-D) cells were evaluated by flow cytometry. (A, C) Representative flow cytometric plots of lymphocytes from each group. (B, D) Percentages of the indicated lymphocytes. The data are expressed as the mean ± SD for each group. *p<0.05; **p<0.01.

Moreover, we detected the proportion of central memory T cells (TCMs, CD44^+^CD62L^+^). As shown in Fig 7, the TCM proportion among CD4^+^ T cells (Fig 7A and 7B) or CD8^+^ T cells (Fig 7C and 7D) in the ZI-E group was significantly different from that in the ZI-D and PBS control groups. This result indicated that ZI-E could promote the production of TCMs by CD4^+^ and CD8^+^ T cells.

## Discussion

On November 18, 2016, the WHO announced that ZIKV was no longer a public health emergency of international concern, but it is still a significant and sustained public health problem. The WHO has emphasized that ZIKV remains a major threat to some areas and has stated that it will fight the virus with a long-term strategy. Moreover, ZIKV disease was listed as a "Disease X" by the WHO in the "Research and Development (R&D) Blueprint for Action to Prevent Epidemics" [24]. In February 2018, the WHO listed ZIKV disease as one of the priority study diseases because of the possibility of repeated outbreaks and the lack of specific drugs and vaccines. Therefore, research on the pathogenesis of ZIKV and the development of a safe and effective ZIKV vaccine are still critical for disease prevention and control.

Among the structural proteins of ZIKV, the E protein can induce the host to produce NAbs and mount protective responses. Researchers have used different strategies to construct vaccines, including incorporation of the whole prM-E gene, various truncation mutants of the prM-E gene or introduction of the corresponding Japanese encephalitis virus (JEV) sequence [19,20], which have had different effects. Larocca [20] produced ZIKV DNA vaccines and found that the prM-E DNA vaccine elicited higher E-specific antibody titers than the DNA vaccine with only the E gene. Therefore, in this research, prM-E of ZIKV was chosen as the target protein to construct a RABV vectored vaccine that could induce cellular and humoral immune responses against ZIKV. Although the prM protein was not evaluated, the E protein was detected by WB using anti-ZIKV E antibodies, and the target band of approximately 55 kDa was consistent with other reports [8,25]. This result indicated that prM-E was cleaved to the E protein correctly.

The RABV SRV9 strain was derived from the SAD strain by plaque purification in BHK cells, and it is nonpathogenic to dogs, rats, guinea pigs, deer, and 3-week-old mice [22]. The vector RABV SRV9, which can highly express foreign proteins, was constructed in our lab [22] and has been used in vaccine development [26,27]. Therefore, we chose the SRV9 strain as the vector to construct three recombinant viruses expressing ZIKV prM-E: ZI-D, ZI-E and ZI-F. Compared with ZI-D, ZI-E replaced the TM region of prM-E with the region of RABV G. Confocal immunofluorescence results showed that the localization of ZIKV E protein on the cell membrane infected with ZI-E was more than that of ZI-D. This phenomenon was consistent with the WB results, in which the ZIKV E protein of ZI-E detected was higher than that of ZI-D. These results indicated that TM region replacement with RABV G could contribute to foreign proteins locating on the cell membrane and being packaged on recombinant viruses. The different immune effects might be due to the different proportions of ZIKV E protein on the ZI-D and ZI-E viruses. In addition, compared with ZI-E, ZI-F replaced the signal peptide of prM-E with that of RABV G. Confocal immunofluorescence, WB and immunogold electron microscopy results showed that no ZIKV E protein expressed. This result indicated that signal peptide replacement might influence ZIKV E protein expression. In addition, the recombinant viruses were obtained by suspension culture of BHK cells, and expression of the exogenous protein did not affect the titer of RABV, making this system convenient for large-scale culture of the virus and the production of antigens.

The adjuvant used in the immunization was ISA 201 VG + poly(I:C); these compounds are more effective when used in combination than when used separately [28]. After immunization, the anti-ZIKV IgG antibody lasted for at least 10 weeks. Upon detection of the NAb at week 6, the PRNT_50_ value was greater than 10. There is only one serotype of ZIKV [29], and an E-specific IgG antibody log titer >2.35–3.2 and an NAb titer >10 are considered protective against ZIKV infection [7,17,20]. The ZI-E designed here could induce the production of sufficient antibodies to be protective against ZIKV. Moreover, the IgG2a/IgG1 ratio is usually used in studies to assess the antibody subtype [28,30–32], and the ZIKV-antibody subtype analysis in this study showed that the antibody was Th2 biased (IgG1 biased). The Th2-biased immune response could be advantageous for protection against some viruses. A Th2-biased subtype was also seen with an Ebola virus (EBOV)-RABV vaccine [33], a MERS-CoV BLP vaccine [28] and a recombinant Rift Valley fever virus (RVFV)-RABV vaccine [26]. Flavivirus-immune sera and mAbs have been shown to be capable of markedly increasing antibody-dependent enhancement (ADE) of infections [34]. Using replication-deficient chimpanzee adenovirus vector (ChAdOx1), Lopez-Camacho [35] constructed ChAdOx1 prME ΔTM vaccine (encoding prM-E without TM). The results demonstrated that anti-ZIKV antibodies induced by vaccines did not induce ADE to dengue virus-2 (DENV2). In this article, when ZI-E was constructed, the TM region of prM-E was replaced with the region of RABV-G. Whether the antibodies induced by ZI-E can enhance ADE against DENV will be evaluated in future studies. Moreover, after immunization with the RABV vaccine, humoral immunity against RABV is dominant. The presence of VNA in serum is considered a reliable indicator, and the WHO and OIE recommend a RABV-VNA titer of ≥0.5 IU/ml as adequate to prevent RABV [36,37]. This standard has been applied in many reports [38,39]. Therefore, in this article, only RABV-VNA was detected, and RABV-T cell responses were not assessed. The titer of RABV NAb was greater than 23.38 IU/ml 6 weeks after the first immunization, much higher than the protective limit of 0.5 IU/ml, indicating that they had protective ability against RABV.

It has been indicated that both antibody- and cell-mediated immune responses play roles in protecting adult mice from ZIKV infection [40]. In this study, ZI-E stimulated the proliferation of spleen lymphocytes and promoted the secretion of cytokines. IFN-γ and IL-4 secretion was significantly induced in splenocytes from immunized mice on day 7 post immunization. Other cytokines with adaptive immunomodulatory roles, including IL2, TNF-α, IL-5, IL-6 and IL-10, were also observed in immunized mice, suggesting that both Th1 and Th2 responses were elicited. The Th1/Th2 immune response is important in vaccination. Th1 cells are crucial for cell-mediated immunity and are characterized by the production of IFN-γ, while Th2 cells can promote humoral immunity and secrete IL-4, IL-5 and IL-6 [41]. The secreted cytokines play important roles in the immune response. If an antigen binding to a B cell receptor is not synergistic with the membrane molecules and cytokines expressed by Th cells, an effective immune response cannot be induced [42]. Meanwhile, although the precise role of T cells in ZIKV infection is not very clear, CD8+ T cells have been determined to play a key role in clearing ZIKV from the central nervous system (CNS) [7], and CD4+ T cells could contribute to protection through cytokine production and support maturation of the antibody response [34]. One of the important purposes of vaccine immunization is to activate immune cells and produce immunologic memory. ZI-E enhanced the activation of antigen-specific CD4^+^/CD8^+^ T cells (Fig 6A–6D), and it also promoted the production of TCMs among CD4^+^ and CD8^+^ T cells (Fig 7). TCMs have the ability to proliferate and produce IL-2, and they play an important role in immune protection [43]. In addition, TCMs have a strong proliferation ability and long life cycles, so they can provide long-term protection [44]. In addition, ZI-E enhanced the activation and maturation of B cells (Fig 6E and 6F), which could further promote the secretion of cytokines and increase antibody levels.

In one modeling study combining multiple factors, researchers determined that many countries across Africa and the Asia-Pacific region are vulnerable to ZIKV disease [45]. Rabies is also a threat to human health in many regions [46,47], including India and China. Therefore, the recombinant viruses designed in this study could also potentially be used as ZIKV-RABV binary vaccines in areas where both ZIKV and RABV are threats.

## References

[1] Dick GW, Kitchen SF, Haddow AJ. Zika virus. I. Isolations and serological specificity. Trans R Soc Trop Med Hyg. 1952;46(5):509–20. Epub 1952/09/01. doi: 10.1016/0035-9203(52)90042-4 . 12995440

[2] WHO. https://wwwwhoint/emergencies/diseases/zika/zika-epidemiology-update-july-2019pdf?ua=1.

[3] Shan C, Xie X, Barrett AD, Garcia-Blanco MA, Tesh RB, Vasconcelos PF, et al. Zika Virus: Diagnosis, Therapeutics, and Vaccine. ACS Infect Dis. 2016;2(3):170–2. Epub 2016/09/14. doi: 10.1021/acsinfecdis.6b00030 . 27623030

[4] Masmejan S, Musso D, Vouga M, Pomar L, Dashraath P, Stojanov M, et al. Zika Virus. Pathogens. 2020;9(11). Epub 2020/11/01. doi: 10.3390/pathogens9110898 ; PubMed Central PMCID: PMC7692141. 33126413 PMC7692141

[5] Kim E, Erdos G, Huang S, Kenniston T, Falo LD Jr., Gambotto A. Preventative Vaccines for Zika Virus Outbreak: Preliminary Evaluation. EBioMedicine. 2016;13:315–20. Epub 2016/10/09. S2352-3964(16)30451-0 [pii] doi: 10.1016/j.ebiom.2016.09.028 ; PubMed Central PMCID: PMC5264651. 27717627 PMC5264651

[6] Abbink P, Larocca RA, De La Barrera RA, Bricault CA, Moseley ET, Boyd M, et al. Protective efficacy of multiple vaccine platforms against Zika virus challenge in rhesus monkeys. Science. 2016;353(6304):1129–32. Epub 2016/08/06. doi: 10.1126/science.aah6157 [pii]. ; PubMed Central PMCID: PMC5237380. 27492477 PMC5237380

[7] Perez P, M QM, Lazaro-Frias A, Jimenez de Oya N, Blazquez AB, Escribano-Romero E, et al. A Vaccine Based on a Modified Vaccinia Virus Ankara Vector Expressing Zika Virus Structural Proteins Controls Zika Virus Replication in Mice. Sci Rep. 2018;8(1):17385. Epub 2018/11/28. doi: 10.1038/s41598-018-35724-6 [pii]. ; PubMed Central PMCID: PMC6255889. 30478418 PMC6255889

[8] Li A, Yu J, Lu M, Ma Y, Attia Z, Shan C, et al. A Zika virus vaccine expressing premembrane-envelope-NS1 polyprotein. Nat Commun. 2018;9(1):3067. Epub 2018/08/05. doi: 10.1038/s41467-018-05276-4 [pii]. ; PubMed Central PMCID: PMC6076265. 30076287 PMC6076265

[9] Barba-Spaeth G, Dejnirattisai W, Rouvinski A, Vaney MC, Medits I, Sharma A, et al. Structural basis of potent Zika-dengue virus antibody cross-neutralization. Nature. 2016;536(7614):48–53. Epub 2016/06/25. doi: 10.1038/nature18938 [pii]. . 27338953

[10] Cugola FR, Fernandes IR, Russo FB, Freitas BC, Dias JL, Guimaraes KP, et al. The Brazilian Zika virus strain causes birth defects in experimental models. Nature. 2016;534(7606):267–71. Epub 2016/06/10. doi: 10.1038/nature18296 [pii]. ; PubMed Central PMCID: PMC4902174. 27279226 PMC4902174

[11] Li XF, Dong HL, Wang HJ, Huang XY, Qiu YF, Ji X, et al. Development of a chimeric Zika vaccine using a licensed live-attenuated flavivirus vaccine as backbone. Nat Commun. 2018;9(1):673. Epub 2018/02/16. doi: 10.1038/s41467-018-02975-w [pii]. ; PubMed Central PMCID: PMC5813210. 29445153 PMC5813210

[12] Gomme EA, Wanjalla CN, Wirblich C, Schnell MJ. Rabies virus as a research tool and viral vaccine vector. Adv Virus Res. 2011;79:139–64. Epub 2011/05/24. doi: 10.1016/B978-0-12-387040-7.00009-3 [pii]. . 21601047 PMC7150175

[13] Abreu-Mota T, Hagen KR, Cooper K, Jahrling PB, Tan G, Wirblich C, et al. Non-neutralizing antibodies elicited by recombinant Lassa-Rabies vaccine are critical for protection against Lassa fever. Nat Commun. 2018;9(1):4223. Epub 2018/10/13. doi: 10.1038/s41467-018-06741-w [pii]. ; PubMed Central PMCID: PMC6181965. 30310067 PMC6181965

[14] da Fontoura Budaszewski R, Hudacek A, Sawatsky B, Kramer B, Yin X, Schnell MJ, et al. Inactivated Recombinant Rabies Viruses Displaying Canine Distemper Virus Glycoproteins Induce Protective Immunity against Both Pathogens. J Virol. 2017;91(8). Epub 2017/02/06. e02077-16 [pii] doi: 10.1128/JVI.02077-16 [pii]. ; PubMed Central PMCID: PMC5375678. 28148801 PMC5375678

[15] Wirblich C, Coleman CM, Kurup D, Abraham TS, Bernbaum JG, Jahrling PB, et al. One-Health: a Safe, Efficient, Dual-Use Vaccine for Humans and Animals against Middle East Respiratory Syndrome Coronavirus and Rabies Virus. J Virol. 2017;91(2). Epub 2016/11/04. doi: 10.1128/JVI.02040-16 ; PubMed Central PMCID: PMC5215356. 27807241 PMC5215356

[16] Willet M, Kurup D, Papaneri A, Wirblich C, Hooper JW, Kwilas SA, et al. Preclinical Development of Inactivated Rabies Virus-Based Polyvalent Vaccine Against Rabies and Filoviruses. J Infect Dis. 2015;212 Suppl 2:S414–24. Epub 2015/06/13. doi: 10.1093/infdis/jiv251 ; PubMed Central PMCID: PMC4564550. 26063224 PMC4564550

[17] Yang M, Lai H, Sun H, Chen Q. Virus-like particles that display Zika virus envelope protein domain III induce potent neutralizing immune responses in mice. Sci Rep. 2017;7(1):7679. Epub 2017/08/11. doi: 10.1038/s41598-017-08247-9 [pii]. ; PubMed Central PMCID: PMC5550446. 28794424 PMC5550446

[18] Dowd KA, Ko SY, Morabito KM, Yang ES, Pelc RS, DeMaso CR, et al. Rapid development of a DNA vaccine for Zika virus. Science. 2016;354(6309):237–40. Epub 2016/10/07. science.aai9137 [pii] doi: 10.1126/science.aai9137 ; PubMed Central PMCID: PMC5304212. 27708058 PMC5304212

[19] Barouch DH, Thomas SJ, Michael NL. Prospects for a Zika Virus Vaccine. Immunity. 2017;46(2):176–82. Epub 2017/02/24. S1074-7613(17)30040-7 [pii] doi: 10.1016/j.immuni.2017.02.005 ; PubMed Central PMCID: PMC5357134. 28228277 PMC5357134

[20] Larocca RA, Abbink P, Peron JP, Zanotto PM, Iampietro MJ, Badamchi-Zadeh A, et al. Vaccine protection against Zika virus from Brazil. Nature. 2016;536(7617):474–8. Epub 2016/06/30. nature18952 [pii] doi: 10.1038/nature18952 ; PubMed Central PMCID: PMC5003703. 27355570 PMC5003703

[21] Krol E, Brzuska G, Szewczyk B. Production and Biomedical Application of Flavivirus-like Particles. Trends Biotechnol. 2019;37(11):1202–16. Epub 2019/04/21. doi: 10.1016/j.tibtech.2019.03.013 . 31003718

[22] Wang H, Jin H, Feng N, Zheng X, Li L, Qi Y, et al. Using rabies virus vaccine strain SRV9 as viral vector to express exogenous gene. Virus Genes. 2015;50(2):299–302. Epub 2015/03/01. doi: 10.1007/s11262-014-1160-y . 25724175

[23] Blaney JE, Wirblich C, Papaneri AB, Johnson RF, Myers CJ, Juelich TL, et al. Inactivated or live-attenuated bivalent vaccines that confer protection against rabies and Ebola viruses. J Virol. 2011;85(20):10605–16. Epub 2011/08/19. doi: 10.1128/JVI.00558-11 ; PubMed Central PMCID: PMC3187516. 21849459 PMC3187516

[24] Barrett ADT. Developing Zika vaccines: the lessons for disease X. Genome Med. 2018;10(1):47. Epub 2018/06/28. doi: 10.1186/s13073-018-0561-2 [pii]. ; PubMed Central PMCID: PMC6019790. 29945653 PMC6019790

[25] Boigard H, Alimova A, Martin GR, Katz A, Gottlieb P, Galarza JM. Zika virus-like particle (VLP) based vaccine. PLoS Negl Trop Dis. 2017;11(5):e0005608. Epub 2017/05/10. doi: 10.1371/journal.pntd.0005608 ; PubMed Central PMCID: PMC5436897. 28481898 PMC5436897

[26] Zhang S, Hao M, Feng N, Jin H, Yan F, Chi H, et al. Genetically Modified Rabies Virus Vector-Based Rift Valley Fever Virus Vaccine is Safe and Induces Efficacious Immune Responses in Mice. Viruses. 2019;11(10). Epub 2019/10/11. E919 [pii] doi: 10.3390/v11100919 [pii]. ; PubMed Central PMCID: PMC6832564. 31597372 PMC6832564

[27] Li E, Yan F, Huang P, Chi H, Xu S, Li G, et al. Characterization of the Immune Response of MERS-CoV Vaccine Candidates Derived from Two Different Vectors in Mice. Viruses. 2020;12(1). Epub 2020/01/24. doi: 10.3390/v12010125 ; PubMed Central PMCID: PMC7019946. 31968702 PMC7019946

[28] Li E, Chi H, Huang P, Yan F, Zhang Y, Liu C, et al. A Novel Bacterium-Like Particle Vaccine Displaying the MERS-CoV Receptor-Binding Domain Induces Specific Mucosal and Systemic Immune Responses in Mice. Viruses. 2019;11(9). Epub 2019/09/01. E799 [pii] doi: 10.3390/v11090799 [pii]. ; PubMed Central PMCID: PMC6784119. 31470645 PMC6784119

[29] Dowd KA, DeMaso CR, Pelc RS, Speer SD, Smith ARY, Goo L, et al. Broadly Neutralizing Activity of Zika Virus-Immune Sera Identifies a Single Viral Serotype. Cell Rep. 2016;16(6):1485–91. Epub 2016/08/03. S2211-1247(16)30980-9 [pii] doi: 10.1016/j.celrep.2016.07.049 ; PubMed Central PMCID: PMC5004740. 27481466 PMC5004740

[30] Kurup D, Wirblich C, Feldmann H, Marzi A, Schnell MJ. Rhabdovirus-based vaccine platforms against henipaviruses. J Virol. 2015;89(1):144–54. Epub 2014/10/17. doi: 10.1128/JVI.02308-14 ; PubMed Central PMCID: PMC4301098. 25320306 PMC4301098

[31] Kurup D, Wirblich C, Ramage H, Schnell MJ. Rabies virus-based COVID-19 vaccine CORAVAX induces high levels of neutralizing antibodies against SARS-CoV-2. NPJ Vaccines. 2020;5:98. Epub 2020/10/23. doi: 10.1038/s41541-020-00248-6 ; PubMed Central PMCID: PMC7568577. 33088593 PMC7568577

[32] Xu S, Jiao C, Jin H, Li W, Li E, Cao Z, et al. A Novel Bacterium-Like Particle-Based Vaccine Displaying the SUDV Glycoprotein Induces Potent Humoral and Cellular Immune Responses in Mice. Viruses. 2019;11(12). Epub 2019/12/15. doi: 10.3390/v11121149 ; PubMed Central PMCID: PMC6950126. 31835785 PMC6950126

[33] Blaney JE, Marzi A, Willet M, Papaneri AB, Wirblich C, Feldmann F, et al. Antibody quality and protection from lethal Ebola virus challenge in nonhuman primates immunized with rabies virus based bivalent vaccine. PLoS Pathog. 2013;9(5):e1003389. Epub 2013/06/06. doi: 10.1371/journal.ppat.1003389 [pii]. ; PubMed Central PMCID: PMC3667758. 23737747 PMC3667758

[34] Pierson TC, Graham BS. Zika Virus: Immunity and Vaccine Development. Cell. 2016;167(3):625–31. Epub 2016/10/22. S0092-8674(16)31253-3 [pii] doi: 10.1016/j.cell.2016.09.020 ; PubMed Central PMCID: PMC5074878. 27693357 PMC5074878

[35] Lopez-Camacho C, Abbink P, Larocca RA, Dejnirattisai W, Boyd M, Badamchi-Zadeh A, et al. Rational Zika vaccine design via the modulation of antigen membrane anchors in chimpanzee adenoviral vectors. Nat Commun. 2018;9(1):2441. Epub 2018/06/24. doi: 10.1038/s41467-018-04859-5 ; PubMed Central PMCID: PMC6015009. 29934593 PMC6015009

[36] Shiota S, Mannen K, Matsumoto T, Yamada K, Yasui T, Takayama K, et al. Development and evaluation of a rapid neutralizing antibody test for rabies. J Virol Methods. 2009;161(1):58–62. Epub 2009/06/02. doi: 10.1016/j.jviromet.2009.05.018 . 19481115

[37] WHO Expert Consultation on rabies. World Health Organ Tech Rep Ser. 2005;931:1–88, back cover. Epub 2006/02/21. . 16485446

[38] Zhang W, Cheng N, Wang Y, Zheng X, Zhao Y, Wang H, et al. Adjuvant activity of PCP-II, a polysaccharide from Poria cocos, on a whole killed rabies vaccine. Virus Res. 2019;270:197638. Epub 2019/06/08. doi: 10.1016/j.virusres.2019.06.001 . 31173772

[39] Mittal MK. Revised 4-dose vaccine schedule as part of postexposure prophylaxis to prevent human rabies. Pediatr Emerg Care. 2013;29(10):1119–21;quiz 22–4. Epub 2013/10/03. doi: 10.1097/PEC.0b013e3182a63125 . 24084614

[40] Brault AC, Domi A, McDonald EM, Talmi-Frank D, McCurley N, Basu R, et al. A Zika Vaccine Targeting NS1 Protein Protects Immunocompetent Adult Mice in a Lethal Challenge Model. Sci Rep. 2017;7(1):14769. Epub 2017/11/09. doi: 10.1038/s41598-017-15039-8 [pii]. ; PubMed Central PMCID: PMC5677088. 29116169 PMC5677088

[41] Siel D, Vidal S, Sevilla R, Paredes R, Carvallo F, Lapierre L, et al. Effectiveness of an immunocastration vaccine formulation to reduce the gonadal function in female and male mice by Th1/Th2 immune response. Theriogenology. 2016;86(6):1589–98. Epub 2016/06/28. S0093-691X(16)30219-9 [pii] doi: 10.1016/j.theriogenology.2016.05.019 . 27344434

[42] Vos Q, Lees A, Wu ZQ, Snapper CM, Mond JJ. B-cell activation by T-cell-independent type 2 antigens as an integral part of the humoral immune response to pathogenic microorganisms. Immunol Rev. 2000;176:154–70. Epub 2000/10/24. doi: 10.1034/j.1600-065x.2000.00607.x . 11043775

[43] Champagne P, Ogg GS, King AS, Knabenhans C, Ellefsen K, Nobile M, et al. Skewed maturation of memory HIV-specific CD8 T lymphocytes. Nature. 2001;410(6824):106–11. Epub 2001/03/10. doi: 10.1038/35065118 [pii]. . 11242051

[44] Maggioli MF, Palmer MV, Thacker TC, Vordermeier HM, Waters WR. Characterization of effector and memory T cell subsets in the immune response to bovine tuberculosis in cattle. PLoS One. 2015;10(4):e0122571. Epub 2015/04/17. doi: 10.1371/journal.pone.0122571 [pii]. ; PubMed Central PMCID: PMC4400046. 25879774 PMC4400046

[45] Bogoch II, Brady OJ, Kraemer MUG, German M, Creatore MI, Brent S, et al. Potential for Zika virus introduction and transmission in resource-limited countries in Africa and the Asia-Pacific region: a modelling study. Lancet Infect Dis. 2016;16(11):1237–45. Epub 2016/10/30. doi: 10.1016/S1473-3099(16)30270-5 ; PubMed Central PMCID: PMC5086423 corporation that models global infectious disease threats. MIC, MG, SB, and AGW have received employment income from BlueDot, and IIB has consulted to BlueDot. All other authors declare no competing interests. 27593584 PMC5086423

[46] Knobel DL, Cleaveland S, Coleman PG, Fevre EM, Meltzer MI, Miranda ME, et al. Re-evaluating the burden of rabies in Africa and Asia. Bull World Health Organ. 2005;83(5):360–8. Epub 2005/06/25. S0042-96862005000500012 [pii]/S0042-96862005000500012. PubMed Central PMCID: PMC2626230. doi: /S0042-96862005000500012 15976877 PMC2626230

[47] Gamble L, Gibson A, Mazeri S, de CBBM, Handel I, Mellanby RJ. Development of non-governmental organisation-academic partnership to tackle rabies in Africa and Asia. J Small Anim Pract. 2019;60(1):18–20. Epub 2018/10/10. doi: 10.1111/jsap.12934 . 30298519

